# Current insights on the genetics and mechanisms of MSX1-associated cleft palate

**DOI:** 10.3389/fdmed.2025.1610223

**Published:** 2025-07-07

**Authors:** AC. Myo, R. Raju, J. O. Piña, P. Chattaraj, M. Furukawa

**Affiliations:** Section on Craniofacial Genetic Disorders, *Eunice Kennedy Shriver* National Institute of Child Health and Human Development (NICHD), National Institutes of Health (NIH), Bethesda, MD, United States

**Keywords:** cleft palate, MSX1, palatal development, recent insights, genetics causes, environmental causes, msx1 downstream signaling pathway

## Abstract

Cleft palate, a common congenital anomaly, is characterized by a failure of the palatal shelves to fuse during embryogenesis, resulting in an opening between the oral and nasal cavities. This malformation not only affects facial aesthetics but also significantly impacts speech, feeding, and hearing, necessitating multidisciplinary care from birth through adulthood. The etiology of cleft palate is complex, involving both genetic and environmental factors. Among the numerous genes implicated, Msx1 plays a pivotal role in palatal development. As a transcription factor, Msx1 regulates mesenchymal cell proliferation and epithelial-mesenchymal interactions, processes crucial for proper palatal shelf elevation and fusion. Disruptions in Msx1 expression or function have been directly linked to cleft palate through both animal and human studies, highlighting its significance in palatogenesis. This review focuses on the role of Msx1 in cleft palate, providing a comprehensive overview of its functions and the molecular mechanisms through which it influences palatal development. We examine recent research findings, including studies on Msx1 mutations, signaling pathways, and gene-environment interactions, to elucidate the complex relationship between Msx1 and cleft palate. Moreover, advancing research could establish Msx1 as a fundamental target in the creation of innovative therapeutic strategies for craniofacial disorders. By synthesizing current knowledge, this review aims to provide a deeper understanding of Msx1's role in cleft palate and pave the way for future research and clinical advancements.

## Introduction

Cleft palate is a congenital malformation characterized by an opening between the oral and nasal cavities, resulting from the failure of the palatine processes to fuse during embryonic development (1). This condition can present in isolation or in conjunction with cleft lip, manifesting in various forms such as soft palate, hard palate, submucosal palate, and complete cleft lip and palate. As one of the most common congenital anomalies (2), cleft lip and palate necessitate multidisciplinary, long-term care from birth through adulthood. The condition significantly impacts aesthetics, pronunciation, feeding, swallowing, hearing, and psychology while also imposing a substantial economic burden. Affecting approximately 1 in 700–1,000 live births, cleft palate incidence varies across racial/ethnic groups, with a tendency for higher rates in Asian populations (3, 4).

Common complications include speech difficulties, such as nasal air emission and mispronunciation, feeding challenges leading to nasal regurgitation and nutritional deficiencies, and increased risk for otitis media and hearing loss. Cleft palate is a multifactorial disorder resulting from a complex interplay of genetic and environmental factors. Several genes, including *MSX1*, *TGFB3*, *IRF6*, *TBX1*, and *PAX9* have been identified as contributors to cleft palate. Among them, *Msx1* is particularly crucial as it encodes a transcription factor that regulates the growth and fusion of the palatine processes, making it one of the key determinants of palatal development.

The etiology of cleft palate involves both genetic predispositions and environmental influences, such as smoking, alcohol consumption, and nutritional deficiencies during critical periods of palatal development. This review will focus on recent research investigating the role of Msx1 in cleft palate, particularly its mutations and their involvement in relevant signaling pathways.

## History of cleft palate research

Early studies on cleft palate date back to the 1840s (5, 6). Research expanded to animal models in the early 20th century, including dogs, horses, and chickens (7–9). Around 1950, cortisone-induced cleft palate in mice became a widely used experimental model (10–13). By the 1970s, researchers began identifying cleft palate as a component of various syndromes, such as Duane's Retraction Syndrome, adducted thumbs syndrome, and Pierre Robin sequence (14–16). Subsequent studies explored the role of etiological factors, including nutritional deficiencies, toxicological exposures, drugs, maternal health, and smoking (17–20). Notably, folic acid supplementation was identified as a protective factor against cleft lip and palate (21, 22). These studies collectively demonstrate that cleft palate development is a complex process influenced by multiple genetic and environmental factors.

## Gene family, expression, and functions of MSX1

MSX1 is a homeobox transcription factor containing a highly conserved homeodomain that facilitates DNA binding and regulation of downstream target genes (23). In humans, the MSX gene family comprises MSX1 and MSX2, both of which are expressed in the embryonic craniofacial region and play crucial roles during development (24). MSX1 functions primarily as a transcriptional repressor, modulating key signaling pathways such as BMP4, Wnt, and Shh—pathways essential for craniofacial morphogenesis (25). During embryogenesis, *Msx1*/ msx1 exhibits strong expression in developing craniofacial and dental tissues (26, 27). In the dental mesenchyme, Msx1 regulates cell proliferation (28) and guides tooth morphogenesis (29). In mice, *Msx1* deficiency results in arrested tooth development at the bud stage (30), while in humans, MSX1 mutations are linked to secondary cleft palate and dental agenesis (24, 30–32). Genome-wide association studies (GWAS) have further implicated *MSX1* in the etiology of cleft palate (33). Functional studies across multiple model organisms, including *Drosophila*, chicken, and zebrafish, support an evolutionarily conserved role for MSX genes in craniofacial development (34–37).

## Syndromic manifestations of MSX1 mutations

MSX1/*Msx1* mutations have been identified as a significant genetic contributor to cleft palate, with functional studies confirming its role in palate, tooth, and craniofacial development (24, 30). These mutations are typically loss-of-function variants, including nonsense, frameshift, and missense mutations that result in reduced or abolished transcriptional activity. Many of these pathogenic variants are located within the highly conserved homeodomain, which is essential for DNA binding and gene regulation (38). Notably, phenotypic variability has been observed depending on the specific domain affected by the mutation, with mutations outside the homeodomain sometimes resulting in milder or distinct phenotypes. Zhao et al. found five new MSX1 variations in Chinese families exhibiting autosomal-dominant nonsyndromic oligodontia (38). These comprised three missense variants (Q221P, R224C, S270l), one nonsense variant (G122*), and one frameshift variant (A93Rfs*67). Significantly, 75% of missense variations and 33% of frameshift variants were situated within the highly conserved homeodomain (HD), indicating that missense variants are more prone to arise in evolutionarily limited locations. Conversely, frameshift variations exhibited no such trend, suggesting that their position is unrelated to amino acid conservation (38).

Mutations in MSX1 have been linked not only to isolated cleft palate but also to syndromic conditions characterized by craniofacial and dental anomalies. For example, Wolf-Hirschhorn syndrome (39) and Witkop syndrome (40, 41) exhibit orofacial clefting and tooth development abnormalities, highlighting the broader impact of MSX1 dysfunction in craniofacial biology. Furthermore, population-based studies suggest that certain ethnic groups may carry MSX1 variants that confer increased susceptibility to cleft palate (42), suggesting a role for MSX1 in future personalized risk assessments (Table 1).

**Table 1:** A summary of Msx1 mutation related syndromes and phenotypes of human and mice.

*Msx1* mutation	Human	Mouse
Syndromes	Pierre-Robin syndrome (OMIM#261800) Wolf-Hirschhorn syndrome (OMIM#194190) Wiktop (dental-nail) syndrome (OMIM#189500)	−
Cleft Palate	+	+
Cleft Lip	+	+
Tooth	Tooth agenesis Hypodontia Oligodontia	Tooth agenesis
Tongue	Micrognathia Glossoptosis	−
Ears	−	Abnormalities in the middle ear, including a shorter malleus and a missing processus brevis
Nails	Dysplasia	Defective nail plates
Others	Craniofacial abnormalities Alveolar bone abnormalities	Abnormalities in skull and nasal bone
SATB2	SATB2-associated syndrome (Glass Syndrome) (OMIM #612313) Tooth agenesis & Cleft palate	Cleft palate Craniofacial anomalies

## Recent insights into Msx1-linked cleft palate: current research findings

### Msx1 promoter activity is regulated by SATB2

**Figure 1:**
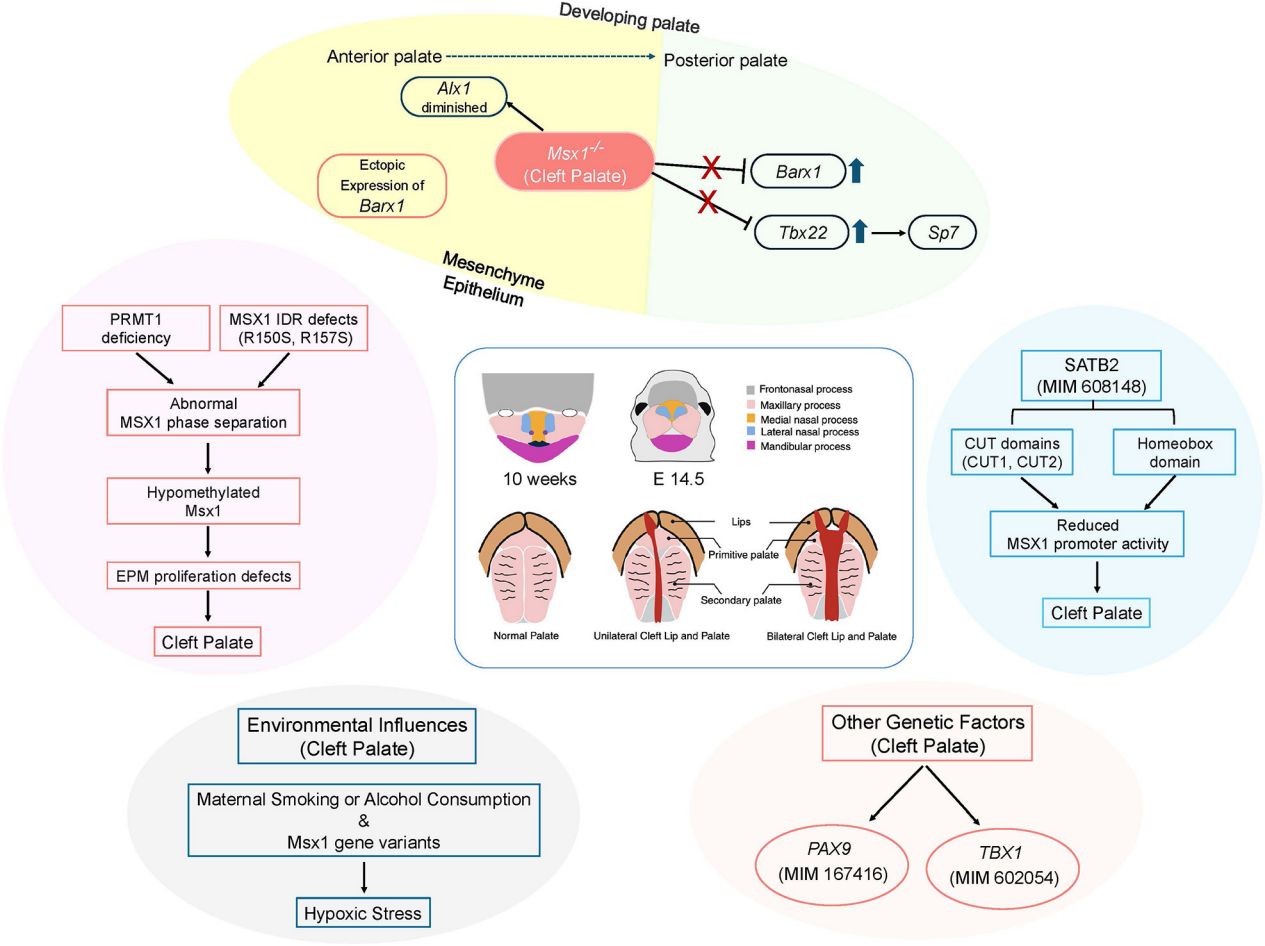
A schematic diagram showing different factors that can lead to cleft palate of human and mice. IDR, intrinsically disordered region; EPM, embryonic palate mesenchyme. Created with BioRender.com.

SATB2 gene plays a major role in various organ and tissue developments such as brain, dental, and jaw (43). SATB2-associated syndrome is characterized by severe intellectual disability, neurodevelopmental disorders, cleft palate, and dental abnormalities (43, 44). In *Satb2^−/−^* mice, reduced *Msx1* expressions were observed at the base of the developing palate (45). Luciferase reporter assay was performed to explore the effects of SATB2 on the Msx1 promoter using wild-type (WT) and variant proteins of SATB2 (A383P, R389C, G392Q, R399H, and F662fs*9). Results showed that SATB2 in WT elevated the activity of the Msx1 promoter, which plays a role in palatogenesis and odontogenesis. However, variants in the CUT1 domain of SATB2 disrupted this transcriptional activation. In immunolocalization studies variants in CUT2 domain of SATB2 localized to cytoplasm instead of nucleus suggesting that the nuclear localization signal of SATB2 resides in the CUT2 domain and that Msx1 promoter due to SATB2 variants may contribute to cleft palate and tooth agenesis in SATB2-associated syndrome (46) (Figure 1).

## MSX1 phase separation leads to the development of cleft palate

Phase separation is a key phenomenon in various biological processes that regulate genetic expressions, signaling, and biochemical reactions (47, 48). This process is often driven by intrinsically disordered regions (IDRs) in proteins (47, 49). In addition to IDRs, posttranslational modifications (PTMs) such as phosphorylation and methylation also play significant roles in regulating phase separation (50) (Figure 1).

The repression domain containing the N-terminal region in MSX1 is a largely unfolded IDR. Protein analysis revealed that under physiological conditions, endogenous MSX1-formed circular droplet-like condensates uniformly distributed in the nucleus in human HEK293 *T* cells, human embryonic palate mesenchyme (EPM) (HEPM), and mouse EPM (MEPM). During osteogenic and chondrogenic differentiation in cultured HEPM, and in mouse palate tissues from gestational day E13.5 to postnatal day P1, MSX1 similarly formed droplet-like condensates in the nucleus indicating its important role in the spatiotemporal regulation of palatal fusion in mouse embryos. It was identified that only *Δ*IDR mutant, which lacks the IDR, exhibited significant defects in nuclear condensate formation, indicating that the IDR is essential for MSX1's droplet-like condensation in cells. This suggests that MSX1 likely undergoes phase separation via its IDR.

PRMT1 is a crucial methyltransferase responsible for R residue methylation in proteins, acting as an upstream regulator of MSX1, thus playing a crucial role in craniofacial development (51). Inhibition of PRMT1 disrupted MSX1 phase separation. Co-localization and reciprocal coimmunoprecipitation of PRMT1 with MSX1 was observed in HEK293T and HEPM cell nuclei. Purified PRMT1 protein was able to directly methylate purified MSX1 protein. Unmethylated MSX1 protein exhibited altered phase separation, with a greater tendency to form condensates compared to PRMT1-methylated MSX1 proteins. The R150S and R157S mutants, which are potential dimethylation sites, showed reduced binding to PRMT1 and had lower methylation levels compared to MSX1-FL (full-length wild-type MSX1). The R150S and R157S mutants exhibited a significant reduction in PRMT1-catalyzed methylation *in vitro*. The abnormal phase separation behavior of R150S and R157S mutants resembled that of MSX1 phase separation when PRMT1 is inhibited. Methylation of the R150S and R157S proteins by PRMT1 significantly reduced their abnormal condensates, indicating that R150 and R157 in the MSX1 IDR, as PRMT1-targeted methylation sites, are crucial for MSX1 phase separation behavior.

The R150S and R157S MSX1 mutants exhibited a reduced proportion of S-phase cells and an increased proportion of G1-phase cells compared to MSX1-FL. These mutants also showed significantly lower expression of both the PCNA gene and protein compared to MSX1-FL, suggesting that the less dynamic MSX1 phase separation caused by R150S and R157S is linked to defects in EPM cell cycle progression and cell proliferation. The increased cell proliferation induced by MSX1-FL was diminished by co-transfection with siPRMT1. Mutations at the MSX1 methylation sites reduced the promoter activities of Tbx22 and Bmp4, important downstream targets of MSX1 in palate development (25). Furthermore, PRMT1-regulated MSX1 methylation and its effects on phase separation influenced the SHH, FGF, BMP, TGFβ, and WNT signaling pathways.

In *prmt1* MO and *msx1* MO zebrafish, there were significantly fewer *egfp*/*phh3* double-positive cells compared to controls, indicating defects in EPM proliferation *in vivo*. These zebrafish also exhibited a higher incidence of cleft palate and defective gel-like condensates. Overexpression of PRMT1 only partially rescued the EPM proliferation defect and palate cleft in *msx1* MO zebrafish. MSX1-FL mRNA rescued the reduced EPM cell proliferation and cleft palate defects in *msx1* MO zebrafish, while MSX1 *Δ*IDR mRNA did not. In mouse models, the knockdown of *prmt1* and *msx1* at E10.5 resulted in a higher incidence of cleft palate compared to controls. Co-injection with adenovirus expressing MSX1-FL (AdFL) promoted palatal fusion and partially rescued the cleft palate caused by shMSX1, while R150S (AdR150S) and R157S (AdR157S) were less effective. This study demonstrates that PRMT1-regulated MSX1 phase separation is a key mechanism underlying cleft palate formation during craniofacial development, with MSX1 phase separation being triggered by its IDR and precisely modulated by PRMT1-mediated dimethylation of R150 and R157 in the MSX1 IDR.

## Hypoxic stress causing cleft palate in Msx1 heterozygosity

Orofacial clefts (comprising cleft anomalies of the lips, palate, and/or facial primordia) can be either syndromic or nonsyndromic malformations with a multifactorial origin, involving both genetic factors and environmental influences (42). Interactions between MSX1 gene variants and maternal smoking or alcohol consumption have been linked to an increased risk of orofacial clefting (52, 53). Additionally, a connection between maternal smoking and MSX1 variants has been shown to elevate the risk for developmental limb malformations (54). Evidence indicates that embryonic hypoxia in the first trimester is associated with craniofacial malformations, including cleft lip and palate (55). While *Msx1* deficiency causes growth defects in the medial nasal process in mouse embryos, it does not affect lip formation (30). To explore potential interactions between *Msx1* deficiency and hypoxia, heterozygous *Msx1* mutant mice were exposed to 10% O_2_ during early lip formation (E10.5 to E12.5). At E15.5, 72% of *Msx1^−/−^* developed cleft lips, either bilateral or unilateral. To model pharmacologically induced hypoxia, pregnant *Msx1^+/−^* females were exposed to varying doses of phenytoin from E10.5 to E11.5. This exposure significantly increased the incidence of cleft lip in *Msx1^−/−^* embryos, reaching 91.7% at the highest dose, with all affected mutants showing a bilateral cleft lip. While *Msx1^+/−^* embryos typically do not have cleft palates, phenytoin treatment caused a higher incidence of cleft palate in these embryos compared to wild-type controls (56). Phenytoin administration also decreased cell proliferation in the palatal processes of both wild-type and *Msx1^+/−^* embryos, and slightly reduced *Bmp4* expression in the anterior palatal process of *Msx1^+/−^* embryos (57).

## *Msx1*/*Tbx22*/*Sp7* axis in the regulation of palatogenesis

The expression of various signaling molecules and transcription factors is tightly regulated along the anteroposterior axis during palate development, with MSX1 playing a specific role in the anterior palate (58) (Figure 1). In a gain-of-function study using *RosaMsx1^Wnt1−Cre^* embryos, abnormal secondary palates were observed (59). These mice exhibited reduced cell proliferation and increased apoptosis in the mesenchyme of the palatal shelves. The size of the maxillary palatine process and palatine bone was notably smaller in *RosaMsx1^Wnt1−Cre^* mice with cleft palate, compared to control mice. The vomer bone was exposed due to the hypoplastic palatal bone, and the presphenoid was also deformed. The expression of RUNX2 was altered, and SP7 was significantly reduced in the palatine process of *RosaMsx1^Wnt1−Cre^* mice. In wild-type mice, *Alx1* was specifically expressed in the mesenchyme of the anterior palatal shelves, but in *Msx1^−/−^* mice, anterior *Alx1* expression was diminished, whereas it increased with *Msx1* overexpression. *Barx1*, expressed in the mesenchyme of the medial and posterior palate in WT embryos, was ectopically expressed in the anterior palate of *Msx1^−/−^* palates, with increased expression in the medial palate. Conversely, *Barx1* expression was significantly reduced in the medial palates of *RosaMsx1^Wnt1−Cre^* mice. These findings suggest that MSX1 is crucial for promoting the expression of anterior-specific genes and inhibiting the expansion of posterior genes into the anterior palate. Similarly, like *RosaMsx1^Wnt1−Cre^* mice, *Tbx22^−/−^* cleft palate mice also showed reduced bone formation in the posterior hard palate. *Tbx22* expression was significantly increased in the medial and posterior palate regions of *Msx1^−/−^* mice but decreased in these regions in *RosaMsx1^Wnt1−Cre^* mice. MSX1 inhibited *Tbx22* promoter activity in a dose-dependent manner. Both protein analysis and RNA-Seq data revealed a significant reduction in *Sp7* in palates with *Msx1* ectopic expression. Knockdown of *Tbx22* expression led to a marked reduction in *Sp7* expression in palatal mesenchymal cells. *Tbx22* overexpression restored SP7 levels in *Msx1*-overexpressing palatal mesenchymal cells. This study shows that *Sp7* is downstream of *Tbx22* in palatal mesenchymal cells and that the *Msx1*/*Tbx22*/*Sp7* axis plays a key role in regulating palate development.

## Conclusion

Recent studies have revealed that Msx1 is not merely an associated factor in cleft palate development but a key transcriptional regulator of craniofacial morphogenesis. As a transcriptional repressor, *Msx1* governs multiple developmental pathways, including cell proliferation, differentiation, and epithelial-mesenchymal interactions. Its mutations or dysregulated expression are linked not only to cleft palate but also to dental anomalies and mandibular underdevelopment. These findings reaffirm that cleft palate is a multifactorial disease rather than a consequence of a single genetic mutation.

However, *Msx1* mutations alone do not account for all cleft palate cases, indicating that complex interactions with genetic background and environmental influences must be further investigated. While rescue studies have shown partial recovery, complete phenotypic correction remains elusive, likely due to Msx1's interplay with diverse signaling pathways.

Additionally, *Msx1* expression must be finely tuned—both excessive and insufficient levels can disrupt normal craniofacial development. Future research should focus on elucidating the upstream and downstream molecular networks regulating *Msx1* to uncover its precise role in craniofacial patterning. Defining how genetic diversity and environmental exposures modulate *Msx1* function will be critical for developing targeted interventions.

Advances in gene-editing technologies, such as CRISPR-Cas9 and single-cell transcriptomics, offer promising avenues for dissecting *Msx1*-related disease mechanisms with unprecedented precision. Ultimately, a deeper understanding of Msx1 will not only advance basic science but also pave the way for innovative strategies in cleft palate prevention, early diagnosis, and personalized therapy. As research progresses, targeting Msx1 could become a cornerstone in the development of novel therapeutic approaches for craniofacial disorders.
