# Correction: Current insights on the genetics and mechanisms of MSX1-associated cleft palate

**DOI:** 10.3389/fdmed.2025.1662124

**Published:** 2025-07-25

**Authors:** AC. Myo, R. Raju, J.O. Piña, P. Chattaraj, M. Furukawa

**Affiliations:** Section on Craniofacial Genetic Disorders, Eunice Kennedy Shriver National Institute of Child Health and Human Development (NICHD), National Institutes of Health (NIH), Bethesda, MD, United States

**Keywords:** cleft palate, MSX1, palatal development, recent insights, genetics causes, environmental causes, msx1 downstream signaling pathway

Incorrect author name

In the author list: In the published article, an author name was incorrectly written as [AC Myo]. The correct spelling is [AC. Myo].

In the citation: In the published article, an author name was incorrectly written as [Myo A]. The correct spelling is [Myo AC].

